# Research Progress on Magneto-Refractive Magnetic Field Fiber Sensors

**DOI:** 10.3390/s23073391

**Published:** 2023-03-23

**Authors:** Linyi Wei, Yang Yu, Dongying Wang, Siyu Yao, Ning Li, Junjie Weng, Shumao Zhang, Jianqiao Liang, Hansi Ma, Junbo Yang, Zhenrong Zhang

**Affiliations:** 1 Key Laboratory of Disaster Prevention and Structural Safety of Ministry of Education, School of Computer, Electronics and Information, Guangxi University, Nanning 530004, China; 2013391126@st.gxu.edu.cn (L.W.);; 2 Guangxi Key Laboratory of Disaster Prevention and Engineering Safety, Guangxi University, Nanning 530004, China; 3 Guangxi Key Laboratory of Multimedia Communications and Network Technology, School of Computer, Electronics and Information, Guangxi University, Nanning 530004, China; 4 College of Sciences, National University of Defense Technology, Changsha 410073, China; yuyang08a@nudt.edu.cn (Y.Y.); wq1211660662@gmail.com (D.W.); yangjunbo@nudt.edu.cn (J.Y.); 5 College of Advanced Interdisciplinary Studies, National University of Defense Technology, Changsha 410073, China; 6 Joint Laboratory for Extreme Conditions Matter Properties, Southwest University of Science and Technology, Mianyang 621010, China

**Keywords:** magnetic field fiber sensors, Magneto-Refractive Effect (MRE), Magneto-Photoelastic Effect (MPE), Magnetic Fluid (MF), Terfenol-D (TbDyFe alloy)

## Abstract

The magnetic field is a vital physical quantity in nature that is closely related to human production life. Magnetic field sensors (namely magnetometers) have significant application value in scientific research, engineering applications, industrial productions, and so forth. Accompanied by the continuous development of magnetic materials and fiber-sensing technology, fiber sensors based on the Magneto-Refractive Effect (MRE) not only take advantage in compact structure, superior performance, and strong environmental adaptability but also further meet the requirement of the quasi-distributed/distributed magnetic field sensing; they manifest potential and great application value in space detection, marine environmental monitoring, etc. Consequently, the present and prevalent Magneto-Refractive Magnetic Field Fiber Sensors (MR-MFSs) are briefly summarized by this paper, proceeding from the perspective of physicochemical properties; design methods, basic performance and properties are introduced systematically as well. Furthermore, this paper also summarizes key fabrication techniques and future development trends of MR-MFSs, expecting to provide ideas and technical references for staff engaging in relevant research.

## 1. Introduction

Magnetic field sensors (namely magnetometers) are an important tool for obtaining magnetic information, with plenty of applications in geophysics, biomedicine, industrial production, and other fields [1,2]. For instance, researchers employ magnetic field sensors to capture low-frequency weak magnetic signals before earthquakes so that pre-warning can be realized [3]. Combined with Magnetic Resonance Imaging (MRI) technology, magnetic field sensors can assist medical staff in disease diagnosis [4]. The employment of Magnetic Prospecting is helpful for workers to acquire the distribution of mineral resources [5]. In the field of marine security, magnetometers are indispensable and vital equipment for military operations, such as anti-mine and anti-submarine operations [6,7]. It can continuously work all day and not be affected by the marine hydrological environment; it also has the advantages of a fast response, high positioning accuracy, and strong concealment [8].

Accompanied by the continuous development of fiber-sensing technology in past decades, magnetic field fiber sensors have been becoming more and more prominent because of their compact structure, superior performance, and strong environmental adaptability, and they can even detect an extremely weak magnetic field down to the fT−level (1 fT = 10^−15^ T) [9]. Compared with single-point-type magnetic field sensors, such as fluxgate [10], giant magnetoresistance [11], and Superconducting Quantum Interferometer Device (SQUID) [12], magnetic field fiber sensors support quasi-distributed/distributed sensing, and can be applied in a dragnet-type search for a large-scale region to improve the capture probability of magnetic anomaly targets [13].

There are kinds of magnetic field fiber sensors in research, such as sensors based on atomic magnetic force [14,15], ampere force [16,17], Faraday optical rotation effect [18], and so forth [19,20,21,22]. Among them, sensors based on the Magneto-Refractive Effect (MRE) can be called, Magneto-Refractive Magnetic Field Fiber Sensors (MR-MFSs). Sensors based on Magnetic Fluid (MF) are a category of MR-MFSs, which are worked by the refractive-index tunability of MF; sensors based on All-Solid Magnetic Materials (ASMMs) are the other, which depend on the Magneto-Photoelastic Effect (MPE) (a refractive-index change effect introduced by strain) with magneto-strictive materials, or the Faraday Optical Rotation Effect (a refractive-index change effect equivalently) with magnetic materials doped.

Sensors based on MF can be employed in geomagnetic detection (μT−level, 1 μT = 10^−6^ T), with low cost and high sensor sensitivity, which have been widely studied in recent years. It is more noteworthy that sensors based on ASMMs are not only compact in structure and convenient for integration, but also support quasi-distributed/distributed sensing. Consequently, sensors based on ASMMs show huge and potential application value in Magnetic Anomaly Detection (MAD) for large-scale operation regions, such as space detection and marine environmental monitoring.

In terms of the above, this paper, with the perspective of MRE, summarizes research progress, design ideas, and properties of MR-MFSs; gives some improvement suggestions; and looks into future development, aiming to provide references for readers and relevant scientific researchers.

## 2. Sensors Based on Magnetic Fluid (MF)

Sensors based on MF generally consist of solid silica-based fiber and MF. MF is a kind of material which has both magnetism and fluidity. It comprises a base carrier liquid, dispersant, and ferromagnetic nanoparticles. MF has a variety of optical properties, such as the Magneto-Electric Directional effect, Magneto-Absorption effect, and MRE [23]. MRE is widely studied in magnetic field fiber sensors based on MF.

**Figure 1:**
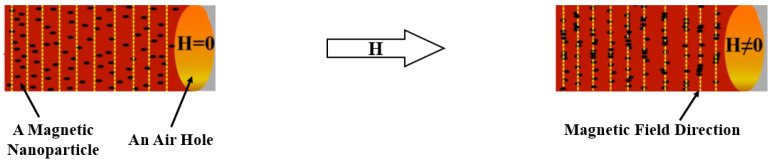
Nanoparticles of MF are rearranged under an external magnetic field (reprinted and adapted with permission from Ref. [24], 2022, Springer Nature).

Some research results provided by Azad et al. [24] indicated that magnetic nanoparticles, which are the efficient composition of MF, will gather and rearrange orderly in the direction parallel with the magnetic field, resulting in the generation of so-called Magnet Chains and the alternation of the refractive index, as shown in Figure 1.

Without a significant Hysteresis effect [25,26], MF has the superparamagnetic property, whose magneto-refractive index tunability can be described by a Langevin function. The Langevin function (Formula (1)) was discovered and given by Chen et al. [27]. In the formula, $n_{\mathrm{MF}(H,T)}$, $n_{S}$, and $n_{0}$ are the current, saturation and critical refractive index of MF, respectively; H and $H_{c,n}$ are the current and critical magnetic field strength, respectively; T is the environment temperature; and α is a fitting parameter.

(1) $$n_{\mathrm{MF}(H,T)}=(n_{S}-n_{0})\left[\coth(\alpha \frac{H-H_{c,n}}{T})-\frac{T}{\alpha (H-H_{c,n})}\right]+n_{0}$$

Zhao et al. [28] adopted the Monte-Carlo method to simulate the arrangement of the magnet chains of MF under different magnetic fields. The research results proved a scientific fact that, when a light propagation direction is parallel with the magnetic field, the refractive index of MF will be increased with the magnetic field’s intensity; on the contrary, the refractive index of MF will be decreased with the magnetic field’s intensity when the light beam is perpendicular to the magnetic field’s direction. This interesting phenomenon can be called the Magneto-Electric Directive effect, which was discovered and proved by Mailfert et al. [29]. Accordingly, vector magnetic field sensors can be fabricated based on this phenomenon.

### 2.1. Based on Fiber Interferometer

Sensors based on the fiber interferometer with MF are a kind of device that can turn the refractive-index change of MF into an optical phase difference, and they have been employed widely owing to their high sensitivity. Such sensors can be divided into four types, that is, sensors based on the Mach−Zehnder Interferometer (MZI), Fabry−Perot Interferometer (FPI), Michelson Interferometer (MI), and Sagnac Interferometer (SI).

#### 2.1.1. Mach−Zehnder Interferometer (MZI)

Sensors based on MZI are generally composed of the fiber cladding (sensing arm) and core (reference arm). When MF changes the Effective Refractive Index (ERI) of a sensing arm, a phase difference between the two arms is produced. By tracking the phase change of the sensor, magnetic field sensing can be realized. However, light in conventional fibers is strictly limited in the fiber core, and the sensing arm cannot be generated. With the help of the up-tapered, offset-core, or other structures, such sensors can be fabricated.

**Figure 2:**
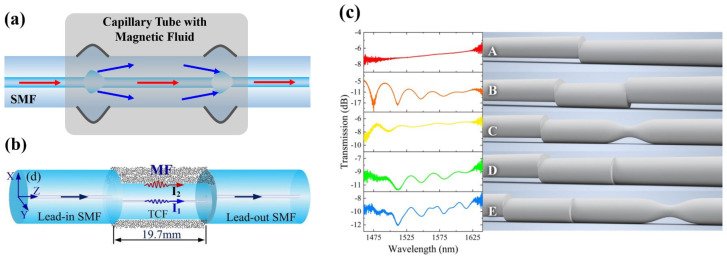
Magnetic field fiber sensors based on (**a**) the up-tapered structure [30], (**b**) the offset-core structure (reprinted with permission from Ref. [35], 2017, Elsevier). (**c**) Some offset–core structures and interference spectrums (reprinted with permission from Ref. [36], 2021, The Optical Society).

The up-tapered structure is generally realized by controlling discharge intensity, axial pushing distance, and other parameters of fiber fusion splicer. Zhang et al. [30] proposed a sensor (Figure 2a) based on Single-Mode Fiber (SMF) and MF, where a proper width of up-tapered makes light beams split and coupled at the fusion point, whose sensitivity is 40.782 pm/Oe. Li et al. [31] introduced MF into the air holes of Photonic Crystal Fiber (PCF). These holes collapsed for over-discharge fusion by operators on purpose. Scattering beams transmitting in the collapsed area increase the Evanescent Field energy of the fiber, making sensor sensitivity up to 72 pm/Oe.

The offset-core structure is generally realized by adjusting the axis and angle deviation during fiber fusion [32]. Such sensors do have a noncircular symmetry in the axial direction (direction of light propagation). In the influence of the magnetic field, the noncircular symmetry will further aggravate the nonuniform aggregation of MF around the fiber, and as a result, such sensors will be more sensitive to refractive index change. For this point, Hu et al. [33] regarded that, a magnetic field sensor with a noncircular symmetric structure would have a good vector property. For instance, Yin et al. [34] proposed a sensor based on the offset-core structure, by using PCF and MF. With the assistance of the asymmetric structure, this sensor possesses four vector-response dominant peaks at 0°, 90°, 180° and 270° in a two-dimensional (2D) magnetic field. Yin et al. [35] further proposed a three-dimensional (3D) vector magnetic field sensor, with a Thin-Core Fiber (TCF) and a similar structure (Figure 2b). This sensor has a low direction error down to ±1.9°. If fiber Evanescent Field energy can be further enhanced, such as by means of increasing the offset-core distance of SMF-TCF, the sensor sensitivity of 22.2 pm/Oe could be further improved. On the other hand, the extinction ratio and interference spectrum are deeply influenced by the offset distance, fusion angle, and fiber shape, as was proved by Hao et al. [36], as shown in Figure 2c. Accordingly, operators should master masterly fusion crafts to fabricate sensors with the offset-core structure, so as to acquire a high-quality and stable spectrum.

**Figure 3:**
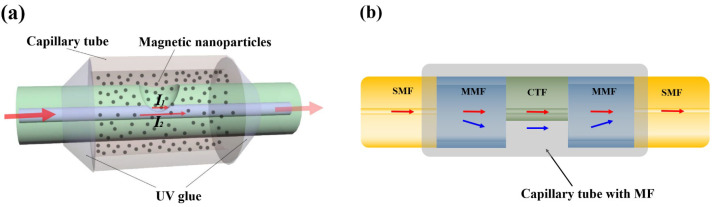
Magnetic field fiber sensors based on the open-cavity structure with (**a**) an SMF (reprinted with permission from Ref. [38], 2022, Elsevier) and (**b**) a CTF [39].

The open-cavity structure is another type of MZI, with the MF acting as a sensing arm and the fiber cladding acting as a reference arm. Under the influence of magnetic field, the ERI and arm difference will be substantially altered, leading to sharp interference spectrum shifting. Hence, such MZIs generally have high sensitivity. Sensors based on the open-cavity structure can be realized by the offset-core structure, which have a longer axis-offset distance. For instance, Liu et al. [37] proposed a sensor based on such a structure with SMFs. MF in its air cavity can modulate the arm difference of MZI to the greatest extent, thus improving the sensor sensitivity up to −1193 pm/Oe. With the assistance of laser burning, such a structure can also be fabricated, but costs are relatively higher. Cai et al. [38] employed a modest-powered nanosecond fiber laser to open a microcavity on an SMF, as shown in Figure 3a. With an ultrahigh scalar sensitivity of 6400 pm/Oe, the proposed sensor also has a good vector sensitivity of 1950 pm/° owing to the design of noncircular symmetry. Moreover, directly employing a fiber with a particular shape to fabricate such a structure is also feasible. Lin et al. [39] exploited Hydrofluoric Acid (HF) to etch a SMF with an offset-core air hole in order to fabricate an open cavity for introducing MF (Figure 3b). The proposed sensor sensitivity is 202.23 pm/Oe, and the sensor also has a good magnetic field vector property. However, the open-cavity structure has a shortcoming in huge optical loss, owing to the high absorption coefficient of MF. Hence, to upgrade the spectrum quality of such sensors, the length of an open cavity and the concentration of MF should both be strictly limited.

#### 2.1.2. Fabry−Perot Interferometer (FPI)

**Figure 4:**
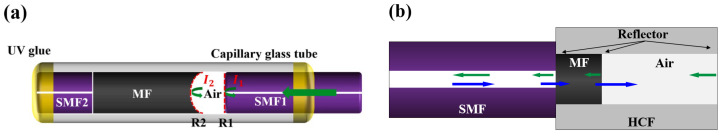
Magnetic field fiber sensors based on (**a**) one-FPI (reprinted with permission from Ref. [40], 2022, Elsevier) and (**b**) treble-FPI [42].

Sensors based on FPI are generally composed of a cavity and a reflector, whose cavity is used to contain the MF. Since they have a relatively high extinction ratio and scalar sensitivity, such sensors are popularly studied by researchers. With the MF volume’s assistance, the sensor sensitivity can be further improved. In terms of the sensor proposed by Wang et al. [40], the length of the air cavity will be altered owing to the MF movement under the magnetic field (Figure 4a). The proposed sensor not only has a high scalar sensitivity of up to 296.1 pm/Oe but also a maximum vector sensitivity of 311.6 pm/°. As mentioned above, if this sensor has a noncircular symmetry in the axial direction, it could have a better vector performance.

The Optical Vernier Effect (OVE) can transform a tiny change into a vernier envelope shift, by an integrated sensor composed of sensing units. With the assistance of the significant shift of a vernier envelope, sensors based on OVE-FPI have higher sensitivity. In the sensor proposed by Zhao et al. [41], incident light is reflected by air and MF successively and recoupled at the output. The generation of OVE makes the proposed sensor sensitivity as high as 1026.02 pm/Oe. Wang et al. [42] also proposed a treble-FPI structure based on MRE and the volume effect of MF (Figure 4b). Under a magnetic field, the light path and ERI of the sensor will be modulated simultaneously, resulting in the proposed sensor sensitivity increasing by 28.16 times (−4219.15 pm/Oe).

#### 2.1.3. Michelson Interferometer (MI) and Sagnac Interferometer (SI)

**Figure 5:**
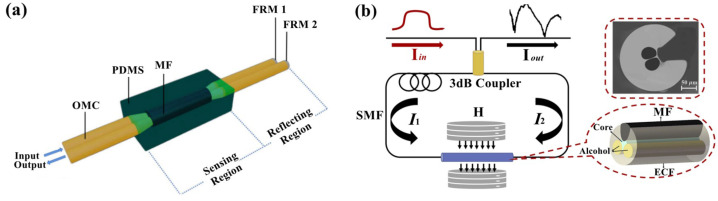
Magnetic field fiber sensors based on (**a**) MI (reprinted with permission from Ref. [43], 2021, The Optical Society) and (**b**) SI (reprinted with permission from Ref. [44], 2022, The Optical Society).

The sensor based on MI is composed of an optical coupler and double reflectors. When lights are transmitted to reflectors, they will be reflected and pass through the coupler again. The optical path and phase of such sensors can be modulated by MF outside. Qin et al. [43] proposed a sensor based on MI (Figure 5a) by using an optical microfiber coupler (OMC) and Faraday Rotation Mirrors (FRMs). The OMC’s high Evanescent Field ratio raises the sensor sensitivity up to 96.8 pm/Oe, but the linear dynamic range is narrow.

The sensor based on SI is also called the Sagnac Loop Mirror, which consists of an optical coupler and a fiber loop. When the MF changes the optical path difference of the loop, the interferometer spectrum will shift. Yu et al. [44] proposed magnetic field sensors based on SI, with the principle of the birefringence effect occurring in an Exposed-Core Fiber (ECF). Owing to the particular structure of the ECF (Figure 5b), MF and alcohol can make enough contact with the Evanescent Field of the ECF, raising sensor sensitivity up to 117 pm/Oe.

### 2.2. Based on Fiber Evanescent Field

Most of the light in conventional fibers is strictly limited in the fiber core, and only a tiny part distributes around the core and attenuates to the cladding, which is called the fiber Evanescent Field. The fiber Evanescent Field can be introduced by many classical methods, such as etching, tapering, micro-bending, side-polished, and so forth. It is really common to exploit MF with MRE to modulate the fiber Evanescent Field in fiber-sensing research.

#### 2.2.1. Fiber Grating (FG)

Fiber Grating (FG) is widely employed in fiber-sensing research, with a relatively high-stable performance. For such sensors based on MF’s refractive index tunability, exploiting FG’s Evanescent Field is a mainstream design method. Many researchers deeply studied this point in the early years. For instance, aiming to leak out FBG’s Evanescent Field and react with MF outside the fiber, Dai et al. [45] exploited HF to corrode FBG’s cladding to reduce its size. Their research results proved a scientific fact: the smaller the FBG diameter is, the larger the Evanescent Field energies and higher the sensor sensitivity are.

In recent years, researchers have paid attention to its magnetic field vector property. Exploiting the asymmetry of refractive index to fabricate vector sensors based on FG is a popular design method, one of which is to employ the difference between fast and slow axis modes’ ERI. For instance, Jiang et al. [46] employed a kind of Thin-Cladding Polarization-Maintaining Fiber Long-Period Fiber Grating (TPMF-LPFG) to propose a sensor based on MF. Owing to different phase velocities, fast and slow axis modes in TPMF-LPFG can both be stimulated. With the assistance of the asymmetry of the refractive index, the proposed sensor has a high vector sensitivity of 72 pm/°. If fiber Evanescent Field energies are stronger, the resolution accuracy of 32.3 μT could be better. Gao et al. [47] employed a kind of FBG with an elliptical core to fabricate a sensor based on MF. By monitoring the change of the fast and slow axis mode’s wavelength interval, a 3D vector magnetic field sensor can be realized, with a vector sensitivity of 15 pm/°.

**Figure 6:**
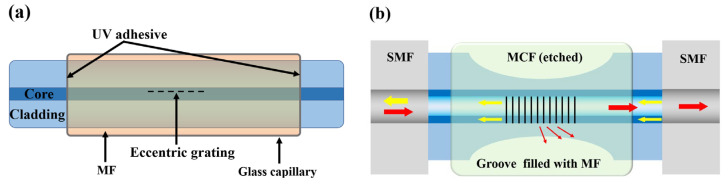
Magnetic field fiber sensors based on (**a**) EFBG [48] and (**b**) MCF [49].

Besides the fast and slow axis’s ERI, directly exploiting a particular FG with an asymmetrical structure is also feasible. For instance, Zhang et al. [48] employed a femtosecond laser to write gratings, and proposed a sensor based on Eccentric Fiber Bragg Grating (EFBG) (Figure 6a). Forward-coupling modes reflected by the gratings interact with MF, which manifest various properties under different magnetic field intensities and directions. Bao et al. [49] wrote gratings on both fiber core and cladding and employed HF to reduce the fiber size, raising the coupling efficiency of the fiber Evanescent Field to MF (Figure 6b). The proposed sensor with a Multi-Claddings Fiber (MCF) has a good response to magnetic field intensity, direction and the environment temperature.

#### 2.2.2. Tapered Fiber (TF)

Tapered Fiber (TF) can be fabricated by HF corrosion, fiber fusion splicer tapering, and flame brush tapering. From the transition to the uniform waist region, the TF diameter gradually decreases. A TF with a tiny enough size can be called a Microfiber, attached with strong Evanescent Field energies and a high environmental sensitivity, which is generally fabricated by flame brush equipment [50,51].

**Figure 7:**
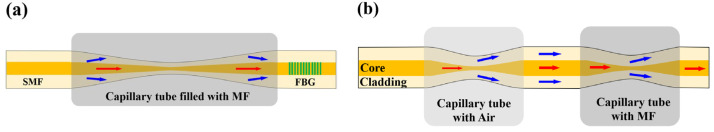
Magnetic field fiber sensors based on (**a**) NATMF (reprinted and adapted with permission from Ref. [52], 2022, IEEE) and (**b**) OVE [55].

In recent years, researchers mainly focus on the TF with high scalar sensitivity, one of which is Non-Adiabatically Tapered Microfiber (NATMF). NATMF is generally fabricated by the two-step tapering method, with the assistance of the fiber fusion splicer and flame brush equipment. Because the foundation mode of NATMF is strongly coupled with cladding and radiation modes, such sensors have high magnetic field sensitivity. For instance, Zhang et al. [52] proposed a magnetic field sensor with a NATMF cascaded with FBG (Figure 7a). This sensor has a sensitivity of 115.9 pm/Oe with a large dynamic range, and can realize magnetic field and temperature simultaneous sensing. Li et al. [53] also proposed a magnetic field sensor with an NATMF. With a larger and stronger Evanescent Field proportion and energies, this sensor has a relatively high sensitivity of 175 pm/Oe and a low magnetic field detection limit of 3.09 Oe. The proposed sensor also has a low-temperature detection limit of 0.471 ℃, which possesses relatively high accuracy and strong robustness.

The second method for improving TF’s sensitivity is based on the dispersion turning point. Some researchers have discovered a scientific fact that optical sensing modes nearby the dispersion turning point do have an ultrahigh sensitivity. Gao et al. [54] studied the relationship between the NATMF’s waist diameters and the dispersion turning point, and proposed a sensor with a high sensitivity of 656.7 pm/Oe.

Furthermore, TF’s sensitivity can also be improved with the assistance of OVE. Gu et al. [55] proposed an integrated sensor (Figure 7b), which consists of a sensing unit filled with MF and a reference unit filled with air. The sensor sensitivity is improved by 4.83 times (−578.2 pm/Oe) owing to OVE. Yuan et al. [56] controlled a Microfiber Coupler (MFC)’s diameter to limit a sensing mode nearby a dispersion turning point. By tracking a vernier envelope, the sensor with an ultrahigh sensitivity of −9785.6 pm/Oe can be realized. Ensuring the output spectrum produced by each separate unit—similar but not equal—is not only the key factor to generate OVE but also a vital guarantee to improve the quality of the vernier envelope and comb-spectrum [57].

Vector magnetic field sensing for TF is also a concern of researchers. Qin et al. [58] exploited an MCF to fabricate a 2D vector magnetic field sensor. Under a magnetic field intensity of 50 Oe, the proposed sensor has a good vector property. Regarding the sensor proposed by Tian et al. [59], two microfibers are fixed to both sides of a capillary glass tube with MF filling. Magnetic field sensing can be realized by tracking its interference spectrum, which is generated by the fiber guided mode and hybrid modes. Owing to the asymmetric design, this sensor has a good vector property, with a low direction error of down to ±1.4°.

However, sensors based on TF are generally fragile, due to the waist diameter being down to the micron or even nanometer level. Thus, some protection and package measures must be considered, such as with the assistance of Polydimethylsiloxane (PDMS) [60].

#### 2.2.3. Surface Plasma Resonance (SPR)

When the Surface Plasmon Resonance (SPR) effect happens, Evanescent Waves in the fiber surface will resonate with plasma waves on a metal surface, generating a Mode-Matching Characteristic Peak, namely the SPR Peak [61]. Because the SPR Peak is especially sensitive to the environment, this effect can fabricate magnetic field fiber sensors with high sensitivity.

**Figure 8:**
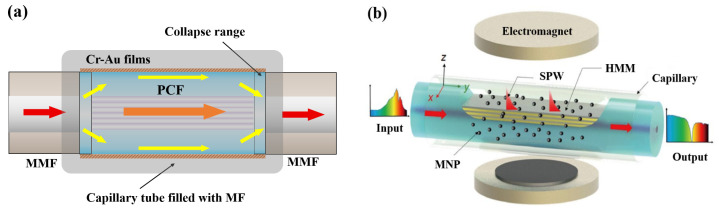
Magnetic field fiber sensors based on SPR with (**a**) MMF-PCF-MMF structure [62] and (**b**) HMMs (reprinted with permission from Ref. [33], 2022, Springer Nature).

The primary issue for fabricating such sensors is how to leak out the fiber Evanescent Field. For this point, a common method is employing a fiber with a large mode field. For instance, Zhu et al. [62] exploited a Multi-Mode Fiber (MMF) and a PCF’s fusion collapse region to induce the fiber Evanescent Field (Figure 8a). Evanescent Waves interact with Cr-Au films and MF, resulting in the generation of SPR. The proposed sensor sensitivity is 442 pm/Oe. Zhou et al. [63] exploited a No-Core Fiber (NCF)’s Evanescent Field to interact with Ag films and MF. The proposed sensor has a sensitivity of 303 pm/Oe. Yao et al. [64] proposed a scheme of depositing Au films on the inner surface of PCF’s air holes to generate SPR, with a high scalar sensitivity of 590 pm/Oe and a low cross-sensitivity of −29.7 pm/℃. However, it is arduous to realize it in practice, regardless of Chemical Vapor Deposition (CVD) or magnetron sputtering.

Besides large-mode field fibers, exploiting a side-polished fiber with Evanescent Field leakage is also feasible. Wang et al. [65] proposed a sensor with Au films deposited on the side-polished surface, which has an ultrahigh sensitivity of 2175 pm/Oe and a good resolution accuracy of 46 nT (1 nT = 10^−9^ T). Moreover, sensors based on the side-polished fiber have a good vector property owing to an asymmetric structure. For instance, Hao et al. [66] employed a kind of wedge-shape fiber to propose a sensor, with a vector sensitivity of 1167 pm/Oe at 0° and −47 pm/Oe at 90°. Lin et al. [67] exploited a half-side SMF’s Evanescent Field to interact with Au films and MF. The proposed sensor has a high vector sensitivity of 1008 pm/Oe at 0° and −336 pm/Oe at 90°, with the assistance of SPR.

On the other hand, the sensitivity of SPR sensors can be further improved by changing the category of metal materials. Regarding the research results provided by Hu et al. [68], Hyperbolic Metamaterials (HMMs) can improve SPR sensor sensitivity by reducing an equivalent dispersion. For this point, Hu et al. [33] further proposed an SPR sensor based on HMMs which consists of alternating Au and ZrO_2_ layers. Evanescent waves leak out from a side-polished surface and interact with HMMs and MF outside the fiber (Figure 8b), elevating a scaler sensitivity up to 1307 pm/Oe. With the assistance of the asymmetric optical field structure, the vector sensitivity was improved by up to 7116 pm/°.

#### 2.2.4. Other Types

In addition to the mainstream types of sensors above, others based on Evanescent Field modulating were also proposed by researchers. In earlier years, some researchers have exploited NCF with its no-core structure and large-mode field property to fabricate sensors based on MF and achieved many excellent research results. For instance, Rao et al. [69] proposed a sensor with NCF. By tracking a leaky guided mode under the refractive-index-matched coupling condition, they realized highly sensitive magnetic field sensing with the highest sensitivity of 633 pm/Oe.

**Figure 9:**
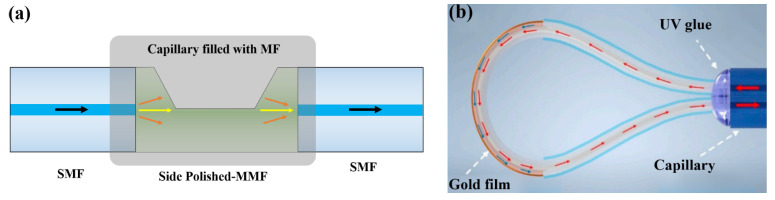
Magnetic fiber sensors based on (**a**) a side-polished fiber [71] and (**b**) a U-shape macro-bending fiber (reprinted with permission from Ref. [75], 2022, MDPI).

Fiber with the side-polished structure can also be used for fabricating sensors based on Evanescent Field modulating. Regarded the sensor proposed by Yu et al. [70], an ECF was applied to make a cavity to fill with MF. Owing to the asymmetric optical field distribution, sensors with the side-polished structure generally have a good vector magnetic field property. For instance, Chen et al. [71] exploited SMFs and side-polished MMF to propose a magnetic field sensor (Figure 9a), with a high vector sensitivity of −5680 pm/°. Xu et al. [72] proposed a sensor with a side-polished structure and MZI. Under the influence of magnetic field, MZI’s arm difference can be modulated by Evanescent Field and MF, resulting in it having different vector properties in different magnetic field directions.

Sensors worked by Whispering-Gallery Mode (WGM) have also been studied in recent years, with advantages in High-Quality Factor and Figure of Merit (FOM) [73], one of which is macro-bending fiber. Macro-bending fiber has a proper bending size, where light in the core overflows into the cladding at a large curvature point, resulting in the generation of cladding modes. On the basis of the sensor with macro-bending fiber proposed by Li et al. [74], Liu et al. [75] deposited gold films on the surface of the fiber by magnetron sputtering (Figure 9b), so that the SPR effect was stimulated. With a high wavelength sensitivity of 974.9 pm/Oe and a good vector property of 546.5 pm/°, this sensor is simple, low cost, and convenient for fabrication.

Sensors based on MF cannot only meet the demand in single-point magnetic field detection, but also support quasi-distributed magnetic field sensing. Ou et al. [76] proposed a sensor based on Evanescent Field modulating, and employed the Fiber Cavity Ringdown (FCRD) technique to monitor the ringdown time and optical intensity change, to realize quasi-distributed magnetic field sensing. The result of the power prediction manifested that 48 detection points can be set at a distance of about 26 km by this scheme. However, the practicability is limited by defects, such as complex light paths and serious optical loss.

From current research results, we see that sensors based on MF mainly support single-point detection. Due to flowability and volatility, the MF in such sensors is difficult to keep stable during transportation. Moreover, the high absorption coefficient of MF seriously weakens optical transmittance, making it hard to use in distributed magnetic fiber sensing. In addition, such sensors have a high cross-sensitivity and narrow linear dynamic range. Owing to the limitations of such shortcomings and issues, sensors based on MF generally exist at the research-laboratory level.

## 3. Sensors Based on All-Solid Magnetic Materials (ASMMs)

Sensors based on ASMMs work according to the Magneto-Photoelastic Effect (MPE), with magneto-strictive materials, or the Faraday Optical Rotation effect (refractive-index change effect equivalently), with some magnetic materials doped. Compared to MF, sensors based on ASMMs not only have compact structures and convenient integration but also support quasi-distributed/distributed sensing; a high and potential applying value is manifested in the field of large-scale magnetic detection, such as undersea target-searching and space exploration.

### 3.1. Based on MPE with Magneto-Strictive Materials

When a fiber is under stress from a magneto-sensitive material, elastic deformation will happen, and then stress-birefringence going with it will change the ERI’s distribution. This phenomenon can be called MPE. When fiber is stressed in the tangential direction, refractive index change can be simply described by the material constant and stress, as shown in Formula (2). For this point, the essence of MPE is a kind of MRE. When it comes to FBG, it has been widely employed in the fabrication of sensors based on MPE, whose Characteristic Peak, ERI, and Grating Period can be described by Formula (3).

(2) Δn=ρσ

(3) $$\lambda_{B}=2n_{eff}\Lambda$$

MPE takes effect by magneto-strictive materials, such as metallic glass (Metglas), TbDyFe alloy (Terfenol-D), etc. Metglas is a kind of amorphous soft magnetic material with a low cost, weak hysteresis effect, and good low-frequency response characteristics. However, it has been less employed at present owing to a weak magneto-strictive effect. Terfenol-D is a new kind of giant magneto-strictive material, namely GMM. Terfenol-D has a high magneto-mechanical coupling coefficient and a strong magneto-strictive effect, which has been widely applied by researchers nowadays.

#### 3.1.1. Ribbon Type

**Figure 10:**
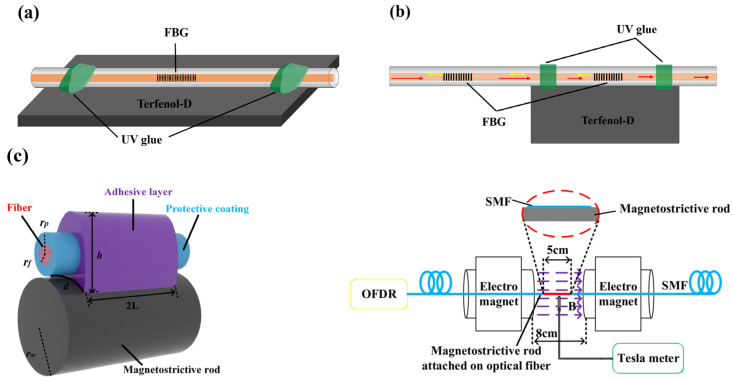
Magnetic field fiber sensors based on the ribbon–type transducer with (**a**) a single-point structure [78], (**b**) a cascaded structure [80], and (**c**) a quasi-distributed structure (reprinted with permission from Ref. [82], 2022, MDPI).

The ribbon type is the most general magneto-optical transducer, which is directly made of the magneto-strictive material with a flat belt or rod shape. Zhan et al. [77] pasted two FBGs on both sides of a Terfenol-D block at an angle, whose sensitivity was 0.877 pm/Oe. With the assistance of Terfenol-D’s magneto-anisotropy, this sensor can realize magnetic field and temperature simultaneous sensing. Kaplan N. et al. [78] proposed a sensor with a Terfenol-D block (Figure 10a), and employed an Optical Frequency–Domain Reflectometer (OFDR) to demodulate sensing signals. The combination of sensing and auxiliary interferometers prompts a spatial resolution accurate within 1 cm. Wu et al. [79] pasted a Terfenol-D rod with the surface of FBGs and employed a dual-frequency optoelectronic oscillator to monitor beat signals. This scheme turns magnetic field signals into frequency, improving the resolution accuracy, shortening the response time, and thus increasing the sensor sensitivity up to −38.4 MHz/Oe.

Sensors based on the ribbon type can also support quasi-distributed magnetic field detection. Regarding the research results provided by Li et al. [80], the sensor with FBGs cascaded manifests a good vector property in a 2D vector magnetic field, which also realizes the simultaneous measurement of the magnetic field and temperature (Figure 10b). The proposed sensor has a compact and simple structure, which can be applied in the quasi-distributed magnetic field sensing research, but the issue of low sensitivity should be solved. Filograno et al. [81] exploited the ribbon–type transducer and a Terfenol-D block to propose a magnetic sensor unit with a highest scalar sensitivity of 1.6 pm/Oe, and it consisted of three orthogonal magnetic field vector probes. A quasi-distributed sensing cascading array was made by connecting these probes one by one, which can not only detect a strong magnetic field nearby MRI equipment but also possesses a good vector property in a 3D space. The proposed sensor can provide data support for workers’ radiation protection. Sun et al. [82] pasted Terfenol-D onto the surface of an SMF and proposed a magnetic field sensor (Figure 10c). Under a magnetic field, the fiber’s strain and ERI will be altered. By tracking Rayleigh Backscattering signals provided by OFDR, the sensor with a sensitivity of 0.505 µε/Oe is fabricated. Owing to its compatibility with the current fiber communication system, this sensor has good feasibility in practice.

#### 3.1.2. Wrapped Type

**Figure 11:**
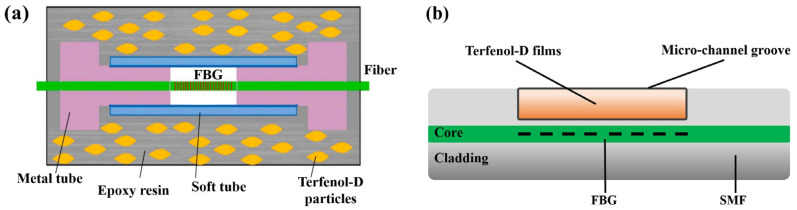
Magnetic field fiber sensors based on the wrapped–type transducer with (**a**) a sensitivity-enhancement structure (reprinted with permission from Ref. [83], 2022, Elsevier) and (**b**) a micro-channel groove [86].

The wrapped type is another common magneto-strictive transducer, and it is made of a sensing fiber wrapped with magneto-strictive powders. Peng et al. [83] made a composite material with Terfenol-D powders mixing glue and proposed a sensor with a sensitivity-enhancement structure (Figure 11a). Under a magnetic field, this structure will concentrate the sensor’s stress. Under a magnetic field, this structure will concentrate the sensor’s stress, raising the sensor sensitivity to 0.983 pm/Oe. Zhang et al. [84] also proposed a sensor with a similar structure, which enhanced the sensor sensitivity by 5.17 times. A research result in earlier years provided by Quintero et al. [85] indicated that sensor sensitivity is impacted by the glues’ category and magneto-strictive powders’ density, and sensor sensitivity can be further improved by imposing some prestress.

Magnetron sputtering is another common method to fabricate the wrapped-type transducer, whose high-speed air pressure makes magneto-strictive powders adhere to the fiber surface. Thus, deposited films with a high adhesion force can be attained. Smith et al. [86] exploited a femtosecond laser to open a groove that was 30 mm long and 20 μm deep to contain Terfenol-D powders (Figure 11b), but the sensitivity was only 0.03 pm/Oe. The research results provided by Yang et al. [87] indicated that, the wrapped-type sensor sensitivity negatively correlates with the diameter. The proposed sensor wrapped by Terfenol-D and Fe/Ni powders has the highest sensitivity. Xu et al. [88] introduced Terfenol-D films into a micro-cantilever structure and turned the ERI signal into resonance. The proposed sensor’s highest sensitivity was improved by up to 40,760 Hz/Oe.

#### 3.1.3. Cylinder Type

The cylinder-type transducer is generally realized by repeatedly winding a sensing fiber on the transducer’s surface. Under a magnetic field, such transducers embedded with magneto-strictive materials will drive the winding fiber by a large margin, significantly altering the interferometer’s optical path difference and enhancing the fiber’s MPE. With the assistance of low-noise demodulation methods, such sensors can realize high-accuracy magnetic detection.

**Figure 12:**
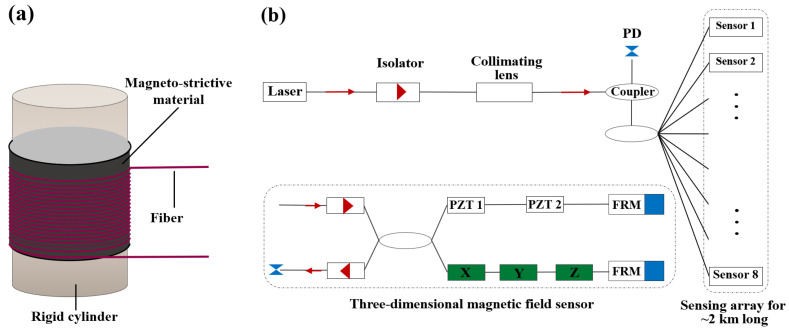
Magnetic field fiber sensors based on the cylinder type transducer with (**a**) a single-point [89] and (**b**) an array sensor [13].

Forty years ago, the U.S. Naval Research Laboratory (NRL) [89] compared magneto-optical transducers among the ribbon, wrapped, and cylinder types (Figure 12a). They concluded that the cylinder type had the best MPE, as it can detect an extremely weak magnetic field down to the pT−level (1 pT = 10^−12^ T). Bucholtz et al. [90] introduced a cylinder–type sensor into MZI, whose arm-length difference can be altered by the influence of the magnetic field. The proposed sensor resolution is $70\;\mathrm{fT}/\sqrt{\mathrm{Hz}}$. Wang et al. [91] also proposed a sensor with a similar transducer, which was combined with a software demodulation algorithm to upgrade a phase-locked amplifier. The proposed sensor can overcome adverse effects from the background magnetic field, ambient temperature and mechanical vibration, which can detect a magnetic field of $100\;\mathrm{pT}/\sqrt{\mathrm{Hz}}$. Chen et al. [92] introduced such sensors into MI, whose arm length can be altered by Metglas material under the magnetic field. The proposed sensor has a resolution of $23\;\mathrm{pT}/\sqrt{\mathrm{Hz}}$.

The successful development of the above sensors provides valuable experience for researching weak magnetic field detection. Sensors with an excellent low-frequency response property are expected to be used in maritime perimeter security in practice. However, corresponding solutions and optimization strategies are still needed for the issues of long-term stability and cross-sensitivity.

Sensors based on the cylinder type can also meet the requirement of quasi-distributed magnetic field sensing. Masoudi et al. [93] wound a SMF on a nickel wire and proposed a magnetic field sensor. By employing Optical Time Domain Reflection (OTDR) to monitor the phase change of backscattered signals, magnetic field sensing can be realized. The proposed sensor has a magnetic field resolution of 30 μT, but the structure is relatively complex, and the light source and demodulation equipment are also expensive. Bucholtz et al. [13] proposed a fiber Magnetic Array System (MARS) which is based on Metglas material and the cylinder type transducer (Figure 12b). Eight single-point sensing units, each of which contains three one-dimensional vector magnetic probes, are employed to consist MARS. A distribution length of about 2 km makes MARS into a quasi-distributed array. Sensing signals are collected and up-converted by MARS so that adverse effects from environmental disturbances and low-frequency noise can be avoided. MARS has a resolution accuracy of up to $200\;\mathrm{pT}/\sqrt{\mathrm{Hz}}$; it was even tested in Norwegian waters for a year and was found to manifest good adaptability. On the other hand, not only should high-resolution accuracy be required for fabricating sensors, but also a Hysteresis effect, cross-sensitivity, environmental adaptability, and other issues should be solved. In addition, when signals are transmitted to a sensor from a long distance, Polarization-Induced Signal Fading phenomenon will be aggravated. This phenomenon needs to be suppressed as much as possible, so as to maintain signal gain at a relatively stable level.

According to current research results, sensors based on MPE are mainly the single-point type still, and some quasi-distributed sensors have been proposed by a few scholars. Due to the cost, uniformity, sensitivity, and other issues of magneto-strictive materials and sensors, it is difficult to fabricate an ultra-long fiber whose whole area is completely sensitive to the magnetic field, aiming to realize distributed sensing. However, compared with sensors based on MF, sensors with MPE have the significant advantages of a stable performance, convenient integration, large dynamic range, and good vector property and support quasi-distributed sensing, owing to the employment of ASMM.

### 3.2. Based on Magnetic Materials Doped

Accompanied by the development of magnetic materials, some novel ASMMs with the property of MRE have been stepping in the view of researchers, such as lead sulfide (PbS) Quantum Dots (PbS-QDs) and Erbium (Er) atoms, whose magnetic moment, Verdet constant (closely related to magneto-optical effect), and other parameters are altered under the magnetic field. Compared with MF and magneto-strictive materials, sensors with novel ASMMs doped not only have more compact structures and stronger practicability owing to an all-fiber design, but are also capable of distributed sensing because the whole fiber is completely sensitive to the magnetic field.

#### 3.2.1. PbS-QDs Doped

PbS-QDs are one kind of ASMMs, which are not oxidized easily and have high thermal stability in natural conditions [94]. Under the impact of magnetic field, owing to the transition and energy of spin electrons changed, PbS-QDs will generate energy-level overlap and asymmetric structures, further resulting in the alteration of the magnetic moment, magnetic susceptibility, and refractive index [95,96,97,98].

**Figure 13:**
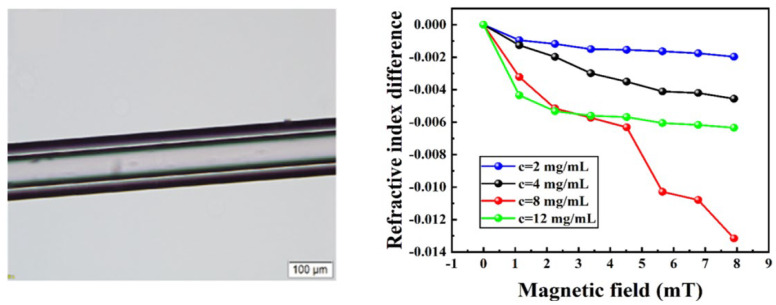
A microscope image and a relationship between different magnetic fields and refractive index with different concentrations based on PbS−QDs doped LCF (reprinted with permission from Ref. [99], 2022, The Optical Society).

Dong et al. [99] employed the density function theory to demonstrate the magneto-refractive property of PbS-QDs and doped colloidal PbS-QDs into a Liquid-Core Fiber (LCF) (Figure 13). When the concentration of PbS-QDs is 8 mg/mL, the proposed sensor has the highest sensitivity of $-1.668\;\times \;10^{-4}$ RIU/Oe. Compared with MF studied by Yang et al. [100], the magneto-refractive sensitivity of PbS-QDs is increased by one order of magnitude. However, the sensor is not all solid-state; the stability and reliability need to be studied further. For this point, Shang et al. [101] conducted a scheme in which PbS-QDs were deposited on the surface of a fiber by the adsorption of an OM. Thus, sensors based on ASMMs can be fabricated by using this method.

#### 3.2.2. Er-Doped

**Figure 14:**
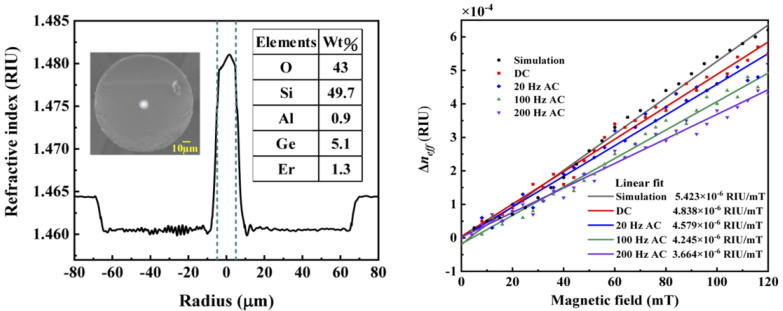
A relationship between the radius and refractive index, element contents in the core; and a relationship between magnetic field and ERI based on an EDF (reprinted with permission from Ref. [103], 2021, The Optical Society).

Er is a rare-earth element with a large spin magnetic moments and a high transition energy level. Er^3+^ has a large number of unpaired electrons at the 4f energy level. These unpaired electrons can transition and split, resulting in Er^3+^ having good magnetic properties [102]. Liu et al. [103] introduced an Er-doped fiber with 1.3 wt% EDF into MZI to fabricate a sensor based on ASMMs, with the highest sensitivity being 4.838 × 10^−7^ RIU/Oe (Figure 14). However, the environmental temperature will change the bond length and angle between atoms in Er-doped fiber, resulting in a refractive index change [104]. Therefore, Er-doped fiber sensors have issues of high cross-sensitivity and serious temperature crosstalk, similar to MF. Accordingly, some measures related to temperature compensation and shield should be considered during sensor fabrication.

Introducing the SPR effect is an efficient measure to elevate sensor sensitivity. Yao et al. [105] proposed an Er-doped PCF sensor based on SPR, whose D-shape not only leaks Evanescent Field interacting with gold films but also ensures the feasibility of the side-polished technique. The proposed sensor has a refractive-index sensitivity of 2.27 × 10^−7^ RIU/Oe.

**Figure 15:**
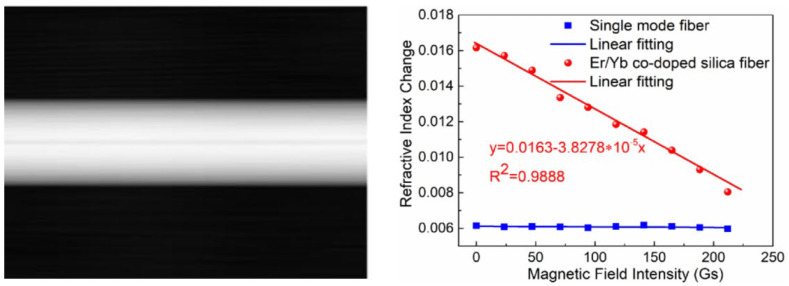
Two-dimensional phase distribution of experimental hologram, and a relationship between magnetic field and ERI based on an EYDF (reprinted with permission from Ref. [108], 2021, IEEE).

Increasing the doping concentrations of Er^3+^ is another way to elevate sensitivity [106]. However, it is inadvisable to increase the concentration of Er^3+^ blindly, for the reason that anions in silicon-based fiber are no probably enough to pair with Er^3+^. A high concentration of an Er^3+^ will cause an uneven distribution of ions and generate a Quenching phenomenon, which weakens the sensor’s performance [107]. Ytterbium ions (Yb^3+^) are capable of inhibiting Er^3+^ clusters and reducing the quenching concentration. Referring to the sensor based on an Er/Yb Co-Doped Fiber (EYDF) proposed by Dong et al. [108], Er with 0.7 wt% and Yb with 0.2 wt% were combined together to form an ASMM-doped fiber (Figure 15.). The proposed sensor, with a good uniformity and stability, has a sensitivity of 3.8279 × 10^−5^ RIU/Oe, which is the same order of magnitude as that of MF [100]. However, the linear dynamic range is only about 200 Oe.

According to current research results, compared to MF, sensors based on ASMMs-doped fiber are more compact and practical with some high magneto-refractive sensitivities. It is vital that the whole area of the fiber is completely sensitive to the magnetic field so that distributed magnetic field sensing can be realized by this design. However, such sensors are still in the preliminary research stage; stability, reliability, cross-sensitivity and other properties need to be further studied by researchers.

## 4. Conclusions and Outlook

In this paper, research progress of MR-MFSs is briefly reviewed. Design ideas and properties of each type of sensor are systematically summarized, and their main performance parameters are shown in Table A1 and Table A2 of Appendix A. Referring to the shortcomings of these sensors, some advice for improvement is offered by this paper.

Sensors based on MF with MRE are the focus of magnetic field sensing research in recent years. Such sensors have high sensitivity, especially for sensors based on the open-cavity MZI, OVE, and SPR. With the assistance of an asymmetric optical field structure, the sensor vector properties can be further improved. However, sensors based on MF have the disadvantages of a high-temperature crosstalk, serious optical loss, narrow dynamic range, and poor stability. Up to now, it has been mainly employed for single-point magnetic field sensing in the stage of laboratory research. Many vital issues in practice should be sorted out in future development, such as temperature compensation, multi-parameter demodulation, transportation, etc.

Sensors based on ASMMs with MRE are majorly fabricated by MPE or fiber doped with magnetic materials. Between them, sensors based on MPE have been developing gradually after decades of development, with stable performance, large dynamic range, and good vector properties. By means of some efficient magneto-optical transducers and low-noise demodulation methods, sensor resolution can be promoted, and extremely weak magnetic detection can be realized. Such sensors are suitable for quasi-distributed magnetic field sensing, but some issues should be sorted out, such as the Hysteresis effect, and Polarization-Induced Signal Fading phenomenon.

Accompanied by the progression of fiber-sensing technology for decades, magnetic materials are also gradually maturing. Some researchers introduced magnetic materials (such as PbS-QDs and Er) into fibers to fabricate magnetic field sensors and attained some preliminary progress. Compared with MF and magneto-strictive materials, sensors based on magnetic materials that are doped have a more compact structure, stronger practicability and higher sensitivity. It is more significant that the whole area of such sensors is completely sensitive to the magnetic field, and distributed sensing can be realized by this design. If an excellent light-transmission property and weak cross-sensitivity are both possessed simultaneously, sensors based on doped ASMMs will play a vital role in magnetic field fiber sensing research.

## Data Availability

Not applicable.

## Abbreviations

Acronyms used in this paper:

**Acronym**	**Definition**
ASMM	All-Solid Magnetic Material
CVD	Chemical Vapor Deposition
ECF	Exposed-Core Fiber
EDF	Er-Doped Fiber
EFBG	Eccentric Fiber Bragg Grating
Er	Erbium
ERI	Effective Refractive Index
EYDF	Er/Yb Co-Doped Fiber
FBG	Fiber Bragg Grating
FCRD	Fiber Cavity Ringdown
FG	Fiber Grating
FOM	Figure of Merit
FPI	Fabry–Perot Interferometer
FRM	Faraday Rotation Mirror
HF	Hydrofluoric Acid
HMM	Hyperbolic Metamaterials
LCF	Liquid-Core Fiber
MAD	Magnetic Anomaly Detection
MARS	Magnetic Array System
MCF	Multi-Claddings Fiber
MF	Magnetic Fluid
MFC	Microfiber Coupler
MI	Michelson Interferometer
MMF	Multi-Mode Fiber
MPE	Magneto-Photoelastic Effect
MRE	Magneto-Refractive Effect
MRI	Magnetic Resonance Imaging
MR-MFS	Magneto-Refractive Magnetic Field Fiber Sensor
MZI	Mach-Zehnder Interferometer
NATMF	Non-Adiabatically Tapered Microfiber
NCF	No-Core Fiber
NRL	Naval Research Laboratory
OFDR	Optical Frequency-Domain Reflectometer
OMC	Optical Microfiber Coupler
OTDR	Optical Time Domain Reflection
OVE	Optical Vernier Effect
PbS-QD	Lead Sulfide Quantum Dot
PCF	Photonic Crystal Fiber
PDMS	Polydimethylsiloxane
PS-FBG	Phase-Shift FBG
SI	Sagnac Interferometer
SMF	Single Mode Fiber
SPR	Surface Plasmon Resonance
SQUID	Superconducting Quantum Interferometer Device
TCF	Thin-Core Fiber
TF	Tapered Fiber
TFG	Titled Fiber Grating
2D	Two-Dimensional
3D	Three-Dimensional
WGM	Whispering-Gallery Mode
Yb	Ytterbium

## Appendix A

**Table A1:** Main performance parameters of magnetic field fiber sensors based on MF.

Sensing Structure	Magnetic Sensitive Materials	Sensitivity	Linear Dynamic Range	Vector Property	Ref
MZI: Up-Tapered Structure	Oil-Based MF	40.782 pm/Oe	0–250 Oe	*	[30]
MZI: Up-Tapered Structure	MF (EMG507)	72 pm/Oe	0–66.6 Oe	*	[31]
MZI: Offset-Core Structure	MF (EMG705)	11.45 pm/Oe 0.179 dB/Oe	20–140 Oe	Yes	[34]
MZI: Offset-Core Structure	MF (EMG705)	22.2 pm/Oe	*	Yes	[35]
MZI: Open-Cavity Structure	MF (EMG509)	−1193 pm/Oe	3–21 Oe	*	[37]
MZI: Open-Cavity Structure	Water-Based MF	6400 pm/Oe	2–16 Oe	Yes	[38]
MZI: Open-Cavity Structure	Water-Based MF	202.23 pm/Oe	0–123 Oe	Yes	[39]
FPI	MF (EMG905)	296.1 pm/Oe	22.6–121.8 Oe	Yes	[40]
FPI: OVE	Oil-Based MF	1026.02 pm/Oe	118.768–166.261 Oe	*	[41]
FPI: OVE	MF	−4219.15 pm/Oe	109.6–125.8 Oe	*	[42]
MI	Water-Based MF	96.8 pm/Oe	0–50 Oe	*	[43]
SI	Water-Based MF	117 pm/Oe	0–55.8 Oe	*	[44]
TPMF-LPFG	MF (EMG605)	−61.8 pm/Oe	0–105 Oe	Yes	[46]
FBG	MF (EMG805)	8.1 pm/Oe	60–220 Oe	Yes	[47]
EFBG	Water-Based MF	0.042 dB/Oe	40–120 Oe	Yes	[48]
MCF	MF	−0.0353 dB/Oe	70–140 Oe	Yes	[49]
NATMF	Water-Based MF	115.9 pm/Oe	0–180 Oe	*	[52]
NATMF	Water-Based MF	175 pm/Oe	0–133.5 Oe	*	[53]
TF	MF (EMG605)	656.7 pm/Oe	0–50 Oe	*	[54]
TF-OVE	Water-Based MF	−578.2 pm/Oe	100–300 Oe	*	[55]
MFC-OVE	MF	−9785.6 pm/Oe	90–97.5 Oe	*	[56]
MFC	Water-Based MF	54.71 pm/Oe	0–60 Oe	Yes	[58]
TF	MF	24.4 pm/Oe	*	Yes	[59]
SPR-PCF	MF (EMG605)	442 pm/Oe	60–240 Oe	*	[62]
SPR-NCF	MF (EMG507)	303 pm/Oe	0–349 Oe	*	[63]
SPR-PCF	MF	590 pm/Oe	50–130 Oe	*	[64]
SPR: Side-Polished PCF	MF	2175 pm/Oe	50–130 Oe	*	[65]
SPR: Wedge-Shape Fiber	MF (EMG605)	1167 pm/Oe	105–155 Oe	Yes	[66]
SPR: Side-Polished Fiber	MF (EMG605)	1008 pm/Oe	20–120 Oe	Yes	[67]
SPR: Side-Polished Fiber	MF (EMG605)	1307 pm/Oe	0–100 Oe	Yes	[68]
NCF	Oil-Based MF	633 pm/Oe	0–60 Oe	*	[69]
ECF	Water-Based MF	−18 pm/Oe	28–73 Oe	*	[70]
Side-Polished Fiber	MF (EMG605)	–53 pm/Oe	0–80 Oe	Yes	[71]
Side-Polished Fiber	MF	24 pm/Oe	340–420 Oe	Yes	[72]
WGM: U-Shape Fiber	Water-Based MF	517 pm/Oe	0–300 Oe	Yes	[74]
WGM-SPR: U-Shape Fiber	MF (EMG605)	974.9 pm/Oe	20–50 Oe	Yes	[75]
FCRD: Side-Polished Fiber	MF (EMG603P)	$1.85\;\times \;10^{-3}$dB/Oe	90–210 Oe	*	[76]

* represents a parameter is not definitely mentioned in related paper.

**Table A2:** Main performance parameters of magnetic field fiber sensors based on ASMMs.

Sensing Structure	Magnetic Sensitive Materials	Sensitivity/ Resolution Accuracy	Linear Dynamic Range	Vector Property	Ref
Ribbon Type: FBGs	Terfenol-D Block	0.877 pm/Oe	80–280 Oe	*	[77]
Ribbon Type: FBGs	Terfenol-D Rod	0.22 pm/Oe	36–700 Oe	*	[78]
Ribbon Type: FBGs	Terfenol-D Rod	−38.4 MHz/Oe	20–70 Oe	*	[79]
Ribbon Type: FBGs	Terfenol-D Blocks	0.2 pm/Oe	0–500 Oe	Yes	[80]
Ribbon Type: FBGs	Terfenol-D Blocks	1.6 pm/Oe	*	Yes	[81]
Ribbon Type: SMF	Fe-Ga Alloy	0.505 µε/Oe	0–150 Oe	*	[82]
Wrapped Type: FBG	Terfenol-D Powders	0.983 pm/Oe	120–1500 Oe	*	[83]
Wrapped Type: FBG	Terfenol-D Powders	0.358 pm/Oe	0–2260 Oe	*	[84]
Wrapped Type: FBG	Terfenol-D Powders	300 μT	*	Yes	[85]
Wrapped Type: FBG	Terfenol-D Films	0.03 pm/Oe	220–320 Oe	*	[86]
Wrapped Type: FBG	Terfenol-D/FeNi Films	0.108 pm/Oe	0–500 Oe	*	[87]
Wrapped Type: FBG	Magneto-Strictive films	40760 Hz/Oe	*	*	[88]
Cylinder Type: SMF	Metglas (2605 S2)	$70\;\mathrm{fT}/\sqrt{\mathrm{Hz}}$ @34 KHz	*	*	[90]
Cylinder Type: SMF	Fe/Ni Alloy	$100\;\mathrm{pT}/\sqrt{\mathrm{Hz}}$ @1 Hz	60–1000 nT	*	[91]
Cylinder Type: SMF	Terfenol-D Rod	$23\;\mathrm{pT}/\sqrt{\mathrm{Hz}}$ @50 Hz	0–100 μT	*	[92]
Cylinder Type: SMF	Nickel	30 μT	1–8 Oe	*	[93]
Cylinder Type: SMF	Metglas (2605 S-2)	$200\;\mathrm{pT}/\sqrt{\mathrm{Hz}}$ @0.1 Hz	*	Yes	[13]
MZI: LCF	PbS-QDs Doped	$-1.668\;\times \;10^{-4}$RIU/Oe	*	*	[99]
MZI: EDF	Er Doped	$4.838\;\times \;10^{-7}$RIU/Oe	0–1200 Oe	*	[103]
SPR: PCF	Er Doped	$2.27\;\times \;10^{-7}$RIU/Oe	50–4050 Oe	*	[105]
MZI: EYDF	Er/Yb Doped	$-{3.8279\;\times \;10}^{-5}$RIU/Oe	0–211.95 Oe	*	[108]

* represents a parameter is not definitely mentioned in related paper.
